# Rethinking Scale-Up of Rehabilitation for Chronic Disease in Low-Resource Settings: Embracing Complexity for Contextual Impact

**DOI:** 10.5334/gh.1360

**Published:** 2024-10-07

**Authors:** Martin Heine, Wayne Derman, Susan Hanekom

**Affiliations:** 1 Julius Global Health, Julius Center for Health Sciences and Primary Care, University Medical Center Utrecht, Utrecht University, Utrecht, The Netherlands; 2 Faculty of Medicine and Health Sciences, Institute of Sport and Exercise Medicine, Stellenbosch University, Cape Town, South Africa; 3 IOC Research Centre, South Africa; 4 Faculty of Medicine and Health Sciences, Division of Physiotherapy, Stellenbosch University, Cape Town, South Africa

**Keywords:** Rehabilitation, Non-Communicable Disease, Health Services Accessibility, Scale-up, Low-Resourced Settings

## Abstract

As the burden of chronic disease and multiple long-term conditions is increasing globally, disproportionally affecting those in low-resourced settings, there is an increasing call to action to scale effective models of care that can assist in mitigating the impact of chronic disease on functioning, activity, societal participation, and health-related quality of life. The aim of this paper is to unpack the contextual factors that have been implicitly and explicitly voiced by researchers reporting on rehabilitation interventions used to manage chronic disease in low-resourced settings. We systematically engaged the literature and applied a reflexive qualitative and systems thinking lens to unpack the contextual factors and their interplay. A total of 40 different contextual factors were derived through an iterative analysis of 144 eligible articles. The identified factors could be packaged into nine system elements or subsystems relevant to the scale-up of rehabilitation for people with chronic disease. The complexity identified encourages a focus on innovative and intersectoral approaches to address the rehabilitation needs in low-resourced settings.

## Introduction

Equitable and quality healthcare is based on the premise that all people have access to interventions and management options that are based on the best available evidence. However, the gap between evidence and clinical practice has been well described in a variety of settings, including for different chronic disease rehabilitation models (e.g., pulmonary rehabilitation, cardiac rehabilitation). To exemplify, a global audit of cardiac rehabilitation programs for patients with ischaemic heart disease revealed that in only 40% of low- and middle-income countries (LMIC), cardiac rehabilitation services are available, roughly translating to one cardiac rehabilitation spot available for every 66 patients with ischaemic heart disease (1,2).

Furthermore, the colliding epidemics of communicable and non-communicable diseases (NCDs) are resulting in an increased prevalence of multimorbidity in LMICs and other vulnerable populations, leading to an increase in the need for rehabilitation globally. However, while rehabilitation is embraced within the universal health coverage (UHC) target of the sustainable development goals as an essential health service, and moreover, access to rehabilitation is considered a human right (3,4), the gap between population needs and availability of rehabilitation is increasing (1). The need for the scale-up of access to rehabilitation services has been recognised through, amongst others (5,6), the adoption of the WHO call for action 2030 and the first ever adoption of a resolution to strengthen rehabilitation in health systems at the 2023 World Health Assembly (7).

However, context in general and low-resource settings specifically may require adaptation to the interventions’ core (e.g., exercise) or peripheral components (e.g., delivery model) to the extent that implementation and effectiveness may be compromised. As such, a clear understanding of the contextual factors and their interconnectedness that could impact the interventions’ success is essential. The aim of this paper, applying a qualitative lens, is therefore to unpack the contextual factors that have been implicitly and explicitly voiced by researchers reporting on rehabilitation interventions used to manage chronic disease in low-resourced settings. Secondly, we aim to draw upon these factors to identify common system elements that may guide thinking towards scale-up and optimization of secondary prevention programs for chronic disease in low-resourced settings.

## Methods

This study uses a mix of different methodological paradigms (8), including a systematic engagement with the literature, adoption of a qualitative analytical lens, and systems thinking principles. This allows for a complex inquiry of systematically identified published literature beyond what could be expected when quantitatively reviewing barriers and facilitators to rehabilitation implementation.

### Identification of literature

We used conventional systematic methods to identify literature in the niche area of exercise-based rehabilitation AND chronic disease populations (NCDs specifically) AND low-resourced settings.

To identify literature, we pragmatically utilized the literature identified through two systematic scoping reviews our team previously published within the context of NCDs, rehabilitation, and low-resourced settings (9,10). Firstly, a scoping review of 60 exercise-based rehabilitation studies for patients with NCDs (Heine et al. 2019). Second, a scoping review on 48 rehabilitation studies in self-reported, low-resourced settings. This review aimed to unravel the concept of low-resourced settings when rehabilitation is concerned (9). Studies included in the latter review (van Zyl et al. 2021), which focused on NCDs (using the same criteria as outlined in Heine et al. 2019), were eligible for inclusion in this review and its qualitative analysis. Subsequently, both reviews were updated (11 March 2022) using conventional procedures for study selection (i.e., screening of title, abstract, and full text by two independent reviewers).

### Qualitative synthesis

We then conducted a reflexive thematic analysis by deductively and inductively coding the text of the selected articles (11), including the introduction up to the discussion section (i.e., entire text body), using the socioecological model (SEM) to guide the deductive analysis. The SEM is particularly useful when determinants of an outcome are nested within different levels of influence (12). Once all articles were coded, we merged all codes into content categories and themes (i.e., key actors) through a series of team meetings. Importantly, this reflexive qualitative synthesis was conducted twice. First, on the set of articles identified within the two previous reviews (i.e., the main analysis). Subsequently, the analysis was replicated in a subset of articles (20%) purposefully selected from the eligible articles identified when updating the search for these two reviews (i.e., validation). Selection was based on potential gaps (e.g., geographical representation, type of chronic condition) in the original analysis and supplemented with a random selection if applicable. This second analysis in a new set of articles allowed to appreciate the level of thematic saturation (i.e., no new were identified) achieved. If no saturation was achieved, a subsequent new set (20%) of articles would have been selected to repeat the analysis for a third time (and so forth) until saturation was achieved (i.e., no new themes identified); however, this was not applicable.

Finally, we developed a preliminary conceptual synthesis of the themes derived from the thematic analysis by reflecting on the interplay between the variables using concept mapping (13). Within the qualitative paradigm of the project, there was less emphasis on the ‘accuracy’ of the data. The research team embraced their diversity (age, gender, profession, and nationality). Both the reflexive thematic analysis and the development of the concept map were completed through a series of team meetings and robust discussions.

### Concepts

#### Rehabilitation

In a healthcare context, rehabilitation is defined as a ‘multimodal, person-centred, collaborative process’, including interventions targeting a person’s ‘capacity and/or contextual factors related to performance’ with the goal of ‘optimizing’ the ‘functioning’ of ’persons with health conditions currently experiencing disability or likely to experience disability, or persons with disability’. Rehabilitation for people with chronic disease, irrespective of the specific chronic health condition (e.g., diabetes, pulmonary disease, cardiovascular disease), is often multi-component (e.g., exercise, education, nutrition, self-management) and inter-disciplinary, aimed at addressing underlying risk factors (e.g., physical inactivity) while optimizing function, activity, participation, and health-related quality of life (14).

#### Scale-up

**Figure 1:**
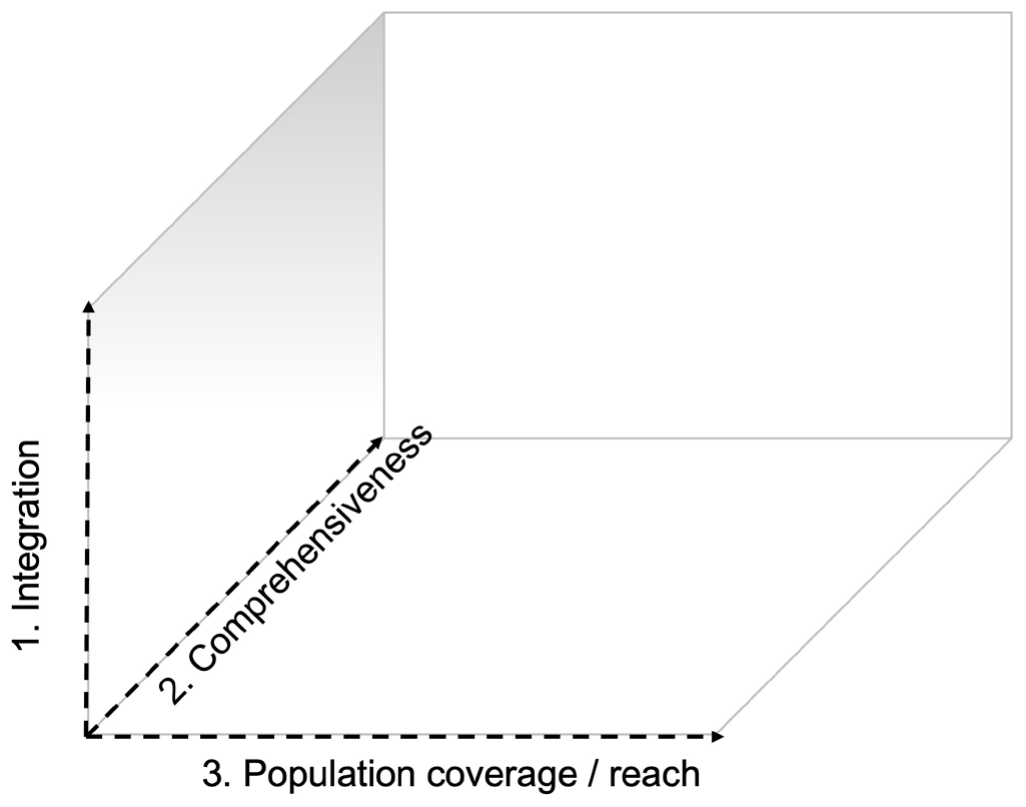
Three-dimensional framework for the scale-up if integrated care is adapted for rehabilitation: (1) strengthening integration within primary care structures, including integrating rehabilitation care for chronic disease within existing programs for communicable disease management (e.g., integrated care for diabetes in patients with HIV/AIDS); (2) increasing the comprehensive nature of existing programs to ensure more core components of rehabilitation are included; and (3) set-up of new programs or program optimization to increase the number of people that have access to rehabilitation services (based upon van Olmen et al. 2021 (16)).

Scale-up is the ‘efforts to increase the impact of health interventions so as to benefit more people and to foster policy and programme development on a sustainable basis’ (15). We’ve tailored the scale-up framework for an integrated care package (16) to a three-dimensional scale-up framework that applies to rehabilitation:

- Strengthening integration within primary care structures, including integrating rehabilitation care for chronic disease within existing programs for communicable disease management (e.g., integrated care for diabetes in patients with HIV/AIDS) and community-based rehabilitation models. Primary care is a key model of care that supports first-contact, accessible, continuous, comprehensive, and coordinated person-focused care (17), often at community level. Strengthening the integration of rehabilitation into primary care has been considered a key strategy for increasing access to rehabilitation (7);
- Increasing the comprehensive nature of existing programs to ensure more core components of rehabilitation are included, including linkage of healthcare and social care to address social determinants of health; and
- Set-up of new programs or program optimization to increase the number of people that have access to rehabilitation services (Figure 1).

The on-the-ground realities in low-resource settings (9) add real layers of complexity to the scale-up of rehabilitation as to mitigate the burden of chronic disease.

## Results

### Study selection for data saturation

A bespoke PRISMA-inspired flowchart is provided in online supplement 1 to showcase the selection process. A full reference list and consolidated overview of all 144 papers (Original [81] and updated [63]) can be found in online supplements 2 and 3, respectively. Thirteen articles (20%) were purposefully selected from articles identified during the search update. These articles were selected based on key areas underrepresented in the main analysis being conducted in a low-income country (n = 5) and patients with cancer (n = 4), supplemented by a final random selection (n = 4).

### Reflexive Thematic analysis

**Figure 2:**
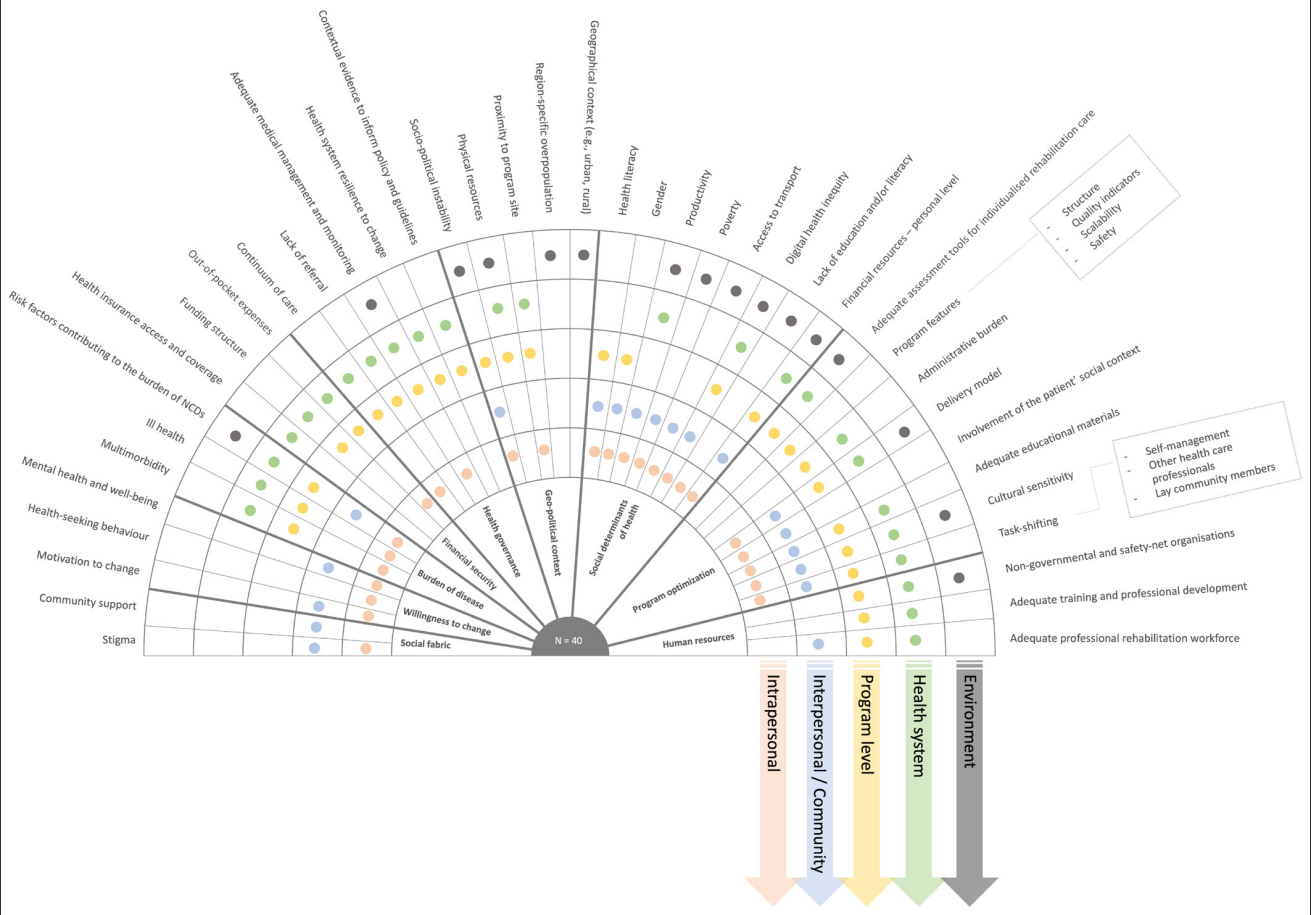
Overview of the 40 themes (outer circle) identified from 169 categories, their relation to the socioecological model (colour coded), and clustered according to nine different subsystems (inner circle; Figure 3). A description of each theme can be found in Table 1.

**Figure 3:**
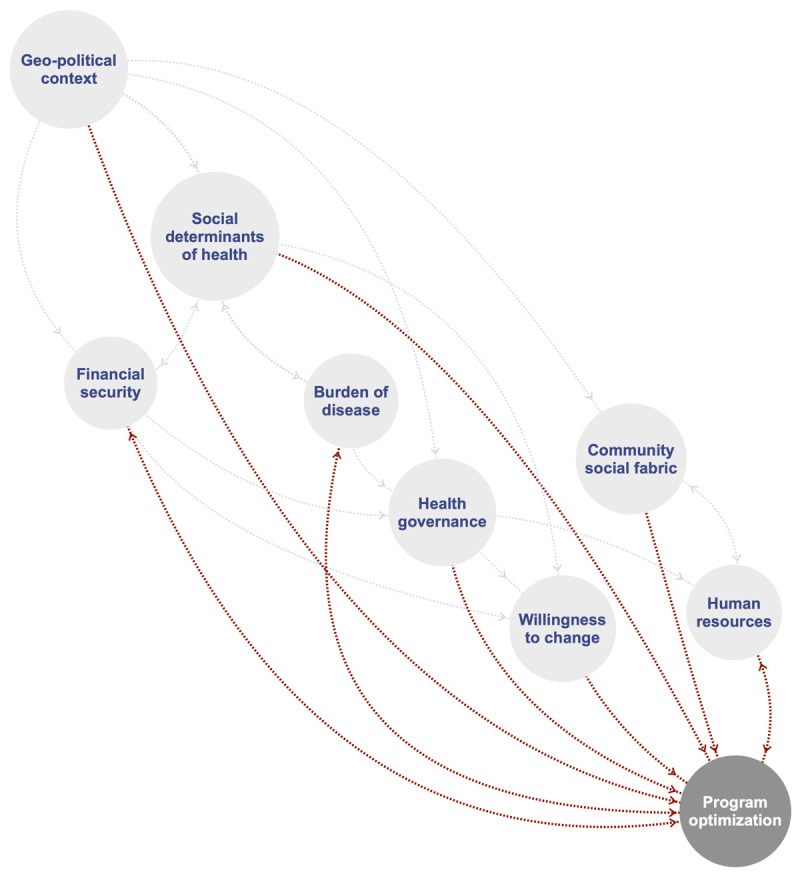
Concept map of mechanisms through which different subsystems may directly (red) or indirectly (grey) inform program optimization and as such promote the integration, reach, and comprehensiveness of rehabilitation for people with chronic disease in low-resourced settings.

The initial analysis resulted in 362 codes, drawn from 81 articles. Subsequently, these codes were merged into 168 categories and 40 themes. In the analysis of the purposefully selected 13 articles, another 51 codes and one additional category were identified (i.e., seasonal fluctuations in resource availability). The single additional category did not warrant additional themes, and therefore the team accepted that data saturation had likely been reached. For the total set of article analyses (n = 81 + 13), six were conducted in low-income countries, 55 in lower-middle income countries, 17 in upper-middle income countries, and 15 in explicit low-resourced settings within high-income countries. A description of each theme, linguistically drawn from the underlying codes and categories, can be found in Table 1. For each theme, the main spheres of influence, according to the socioecological model, are provided (Figure 2). The themes are clustered into ‘sub-systems’ (inner circle, Figure 2) based on the SEM model. The interplay between the nine subsystems that directly or indirectly may inform program optimization to promote program integration, comprehensiveness, and/or reach is depicted in a concept map (Figure 3).

**Table 1:** Description of the forty contextual factors (i.e., themes) identified during the reflexive thematic analysis that may affect successful implementation or scaling of rehabilitation. The theme description is drawn from its underlying codes and categories.


**Access to transport**. Availability of personal transport (e.g., car), availability, affordability, and safety of public transport (e.g., bus, taxi).

**Adequate assessment tools for individualised rehabilitation care**. Tailoring the program content and structure to meet individual patient needs may be essential in achieving comprehensive health benefits. Individualised assessment tools that are simple and accessible and that can inform individualised rehabilitation care are imperative.

**Adequate educational materials**. Educational materials are used to promote health literacy, health-seeking behavior, or in the context of self-management. Adequate education material is personalised, provided in a manner conducive to the patient’s context (including when provided through e-solutions), and tailored in terms of cultural sensitivity, gender, and language. The information needs may shift throughout the continuum of care in relation to the chronicity of the disease.

**Adequate medical management**. Detection of people at risk, routine monitoring, communication between health professionals through the continuum of care, and so forth. Inadequate medical management may affect the health and well-being of the participants, with implications for, amongst others, referral to the rehabilitation team, multimorbidity, and program safety. In some settings, adequate medical management may only be available when insured or with significant out-of-pocket expenses.

**Adequate professional rehabilitation workforce**. Quantity and quality of human resources known to be important in the provision of (multidisciplinary) rehabilitation, including allied health professions, medical oversight of (high-risk) patients, administrative staff, psychologists, nurses, and others. In some settings, tasks are shifted to non-conventional health professionals, lay community members, or an increased focus on self-management.

**Adequate training and professional development**. Continued development of those involved in the rehabilitation process includes knowledge, skills, and competencies of health professionals, but also adequate training when tasks are shifted to lay community members. The training needs may shift in relation to the prevailing risk factors or disease burden.

**Administrative burden**. The time and effort required for program administration and record keeping. Administrative burden, for example, is increased in settings where patient information cannot be easily shared (e.g., paper records) or in settings where patients are difficult to reach (e.g., rapid changing contact details).

**Community support**. A community social fabric where people with chronic disease are socially supported. For instance, related to the stigmatisation of disease and may influence mental health and well-being.

**Contextual evidence to inform policy and guidelines**. High-quality evidence for the cost benefits of rehabilitation in a specific setting can assist in developing context-specific guidelines and policies. The ‘benefits’ need to align with both the patient-perspective and key information required for upscaling and policy initiatives. Protocolised care may improve quality and consistency in rehabilitation programs’ offerings and perceived value.

**Continuum of care**. Non-communicable disease or sequalae of communicable disease are inherently chronic and therefore pose important challenges to providing long-term adequate care across the continuum.

**Cultural sensitivity**. Implementation of rehabilitation is partly dependent on how well the program is aligned with cultural and/or religious features common to the catchment area. For example, in some settings, programs may need to be gender-specific to remove culturally informed barriers to participation. In other settings, the type of exercise therapy needs to be adjusted to the local context, or educational materials need to be developed cognisant of cultural, religious, or language-related factors.

**Delivery model**. Inpatient, outpatient, home-based, telehealth, hybrid, and out-reach were some of the delivery models identified. The delivery model most adequate may depend, amongst others, on the desired program structure, patient population, program safety, geographical context, human resources (including task shifting), funding structure, and digital health equity.

**Digital Health Equity**. Digital health innovation needs to be scalable and accessible to the local demographic and may mean that the latest technological advances are excluded. Challenges may relate to access to information technology, digital literacy, and personal financial resources.

**Financial resources – personal level**. Rehabilitation has the potential to alleviate the impact of disease on financial well-being. Conversely, in the absence of social security measures, work was an important barrier to rehabilitation uptake and adherence. Personal financial resources, in relation to rehabilitation, may also affect the ability to pay for health insurance, out-of-pocket expenses, and transport. Moreover, financial resources may vary with time (e.g., more income during specific seasons) and therefore may affect continuity of care.

**Funding structure**. The way (e.g., government, private) the provision of rehabilitation is funded may affect access, human resources, and scalability, amongst others.

**Gender**. There are significant differences in the barriers experienced to participate in rehabilitation between men and women. For example, there may be cultural/religious barriers that would require women-specific programs, the disease burden and comorbidity profiles may differ, or family responsibilities may hamper participation. Conversely, in some settings, men may be primarily responsible for income generation, which would argue for rehabilitation, yet it also poses an important barrier to participation.

**Geographical context**. Urban and rural are the two most common denotations that speak to the geographic context. Low-resourced settings can be found in both, however, with different features that impact the implementation of rehabilitation. For example, in a rural context, proximity to healthcare providers may be challenging, and hence out-reach programs may be more conducive. Conversely, outreach programs can be challenging in relation to the chronicity of disease. In an urban context, challenges may be related to community safety or violence, to overpopulation or overburdened public health facilities.

**Health insurance**. Affordable health insurance with adequate coverage with increase the implementation of rehabilitation.

**Health literacy**. Various aspects of health literacy influence the implementation of rehabilitation through various mechanisms, including health-seeking behavior. However, health literacy also informs program requirements, education materials, ill health, and multimorbidity. Various social determinants of health (e.g., education, poverty) will affect health literacy levels.

**Health systems’ resilience to change**. The burden of disease in many low- and middle-income countries is shifting from communicable disease to non-communicable disease. The convergence of the two poses a significant threat to health systems that are less resilient to adapt to the different care requirements, which can be exemplified by inadequate funding allocation, professional development, and human resources.

**Health-seeking behavior**. Closely related to health literacy and motivation to change is health-seeking behavior as the active pursuit of care when needed.

**Ill health**. Uncontrolled hypertension and blood glucose are commonly indicated as reasons for non-adherence or barriers to rehabilitation uptake. Imposes a need for thorough risk screening, stratification, and medical oversight prior to and during exercise-based rehabilitation. May impact program safety, needs for medical oversight and ability of the health system to respond to adverse events.

**Involving the patient’s social context**. Programs in which family members join the treatment are considered more enjoyable and motivating. Closely related to social support.

**Lack of referral**. In many settings, referral from the tertiary care facility to either primary or secondary care rehabilitation programs is inconsistent or absent. In the absence of adequate referral structures, the ownership of continued care is shifted to the patient, and therefore the uptake of rehabilitation is increasingly dependent on health literacy and health-seeking behavior, both affected by a series of social determinants prevalent in low-resourced settings. Information technology may reduce the administrative burden associated with referrals where possible.

**Level of education and/or literacy**. Quality education may be one of the key social determinants of health. In the absence of quality education, there are considerable implications for the implementation of rehabilitation, including health literacy, digital health literacy, and the impact on program-specific or educational materials.

**Mental health and well-being**. Poverty, food or financial insecurity, socioeconomic instability, ill health, and other all may affect mental health and well-being. Indirectly, these factors may affect the uptake and adherence to rehabilitation, for instance, through reduced motivation to change.

**Motivation to change**. The insight and motivation that a change is required to improve health. With respect to rehabilitation and chronic disease, this is important for risk factor modification, including physical inactivity, substance use, and nutrition. Such motivation may be expedited in a context where change is encouraged and facilitated.

**Multimorbidity**. The convergence of maternity-related disease, trauma, communicable and non-communicable disease, in conjunction with inadequate medical care, and adverse social determinants of health implies a high level of multimorbidity in low-resourced settings. This affects various aspects related to the implementation of rehabilitation in these settings, amongst others, the scale of rehabilitation needed, access to the program, complexity of patients (and subsequent knowledge and competencies of the program), program structure, and delivery model.

**Non-governmental or safety-net organizations**. In some settings, rehabilitation is provided through non-governmental (e.g., outreach programs) or safety-net organizations (e.g., service-learning-driven programs). The organizations address important service gaps.

**Out-of-pocket expenses**. Monetary expenses associated with receiving care, including over-the-counter medication, but also indirect costs like transport.

**Physical resources**. Physical infrastructure (e.g., buildings) and equipment needed to provide adequate rehabilitation. Factors that may affect physical resources, amongst others, are the geographical context (e.g., rural), funding structure, and sociopolitical stability.

**Poverty**. Lacking enough resources to provide the necessities of life, including access to healthcare and education.

**Productivity**. Beyond financial resources, productivity also entails the ability to participate in meaningful life roles, including family responsibilities, household tasks, social responsibilities, and volunteer work.

**Program features**. There are multiple factors that relate to adequate rehabilitation programs in low-resourced settings that pertain to how these programs are structured (e.g., number of sessions, duration). In part, this may be related to the available resources, including the ability to shift tasks and inform the delivery model. Program structure, disease population, medical management, available human resources, and delivery model may inform program safety and scalability. Program quality can be enhanced by ensuring adequate effective communication structures, safety protocols, as well as structured reporting and monitoring.

**Proximity to program site**. Distance between the patient and nearest rehabilitation program. Can be bridged using task-shifting type rehabilitation models or using eHealth solutions.

**Region-specific overpopulation**. Particularly in urban low-resourced settings, there may be a considerable mismatch between healthcare/rehabilitation needs and healthcare/rehabilitation resources. In some settings (e.g., townships), such needs tend to cluster parallel to poverty, adverse social determinants of health, sociopolitical instability, and others.

**Risk factors contributing to the burden of chronic disease**. In different settings, different risk factors may be more prevalent. This will affect the local burden of disease, yet may also affect program composition, required human and physical resources, feasible task-shifting, skills, and competencies.

**Sociopolitical instability**. The sociopolitical dynamics in some settings can be volatile, including protest action or gang violence, amongst others. These dynamics may impact the implementation of rehabilitation (e.g., access, human resources, safety), as well as the need for rehabilitation programs to remain amphibious to a level of uncertainty at a personal and/or community level.

**Stigma**. A setting in which disease or (components of) rehabilitation are viewed in a negative way. For example, in some settings, women are not expected to participate in moderate-to-vigorous-intensity leisure exercise.

**Task-shifting**. Considering (human) resource constraints, various forms of task shifting emerged from the literature. Firstly, rehabilitation programs strongly focused on self-management. Secondly, tasks conventionally attributed to allied health professionals were shifted to, for example, nursing staff, pharmacists, or other health professionals more likely to be available. Thirdly, there is also considerable drive training and leveraging the use of lay community members for the provision or support of rehabilitation, including peer educators, community health workers, or care navigators.


## Discussion

To our knowledge, this is the first paper to approach the need for upscaling rehabilitation care for chronic disease in low-resourced settings from a holistic and complex lens. This paper outlines 40 critical factors, drawn from multiple data sources, collated into 9 subsystems, that may inform the thinking around scale-up and optimization of rehabilitation in low-resourced settings. We argue that these factors are imperative to the (i) integration of rehabilitation within existing health structures, (ii) the comprehensive nature of the rehabilitation program offered, and when attended to adequately, will likely (iii) increase population coverage, reach, and access. The nine subsystems (Figure 3) provide a template for future research and scale-up initiatives aimed at closing the rehabilitation need versus access gap. Herein, we reflect on the critical factors and subsystems presented in relation to each of the three dimensions of scale-up described.

### Integrated Rehabilitation

The first dimension of scale-up is integration of care within existing health structures. Within the rehabilitation space, we could discern three forms of ‘integration’ that are particularly relevant to address the rehabilitation need. Firstly, integration of rehabilitation care for multiple conditions simultaneously to address multimorbidity or converging burdens of disease (18). Secondly, integration of rehabilitation care with other social domains that therefore can target social determinants of health (19). Thirdly, integration of rehabilitation within primary care structures (20). The value of each of these three becomes apparent when reflecting on the critical factors and subsystems identified in this paper. In many low-resourced settings, multiple burdens of disease collide with human resources (i.e., quantity, adequate training) and health system governance unequipped to tackle the convergence of, for example, communicable and non-communicable disease or diabetes and hypertension, as in our case example (21,22,23,24). Hence, within rehabilitation care for chronic disease, too, we may need to move beyond disease-siloed programs (e.g., cardiac rehabilitation, pulmonary rehabilitation) towards person-centered and goal-oriented care models and provide real-world evidence in their support (25). In providing such support, some of the key questions for rehabilitation in low-resourced settings remain with respect to implementation, adequate monitoring and evaluation to inform policy, cost-benefits, and impactful implementation (26,27).

Furthermore, there is clear evidence that factors outside of healthcare delivery greatly affect an individual’s health and well-being; this is particularly true for low-resourced settings with a high prevalence of adverse social determinants of health. In addition to the impact of social determinants of health on health and well-being, these determinants also directly impact the implementation of rehabilitation care (28,29). Some critical factors and social determinants of health identified in this reflexive thematic analysis were, for instance, health literacy, health-seeking behavior, poverty, productivity, and access to transport. Such social determinants of health may inform both the burden of disease through underlying risk factors ((30)), financial safety net to engage with rehabilitation services, and willingness or ability to make lifestyle changes that can contribute to reduced risk. Hence, rehabilitation-led models of integrated rehabilitation and social care (e.g., case managers) that comprehensively address social determinants of health can make a remarkable difference in the burden of disability and health inequities (19). Awareness of individuals’ social needs, adjustment of care provided, assistance for the individual to have needs met, alignment of efforts with community and social care organizations, and advocacy for changes to infrastructure and policy (both health and social) may facilitate such rehabilitation-led models (19).

Primary care is a model of care that supports first-contact, accessible, continuous, comprehensive, and coordinated person-focused care. The World Health Organization has specifically called for strengthening and scale-up of interventions within primary care in their draft 2023–2030 roadmap on the prevention and control of NCDs (31). Various health systems have approached the integration of rehabilitation into primary care differently, reflecting differences in the organization of care and health governance (32). Six different ways of integrating rehabilitation in primary care have been described, including clinic-based, outreach, self-management, community-based rehabilitation, shared care, and case management (20), Each of which emerges as possible models in relation to the subssystems identified in this reflexive analysis, including the geopolitical context (e.g., rural context informing an outreach model), human resources (e.g., task-shifting, self-management), and health governance (e.g., resilient health systems, referral pathways). The work presented here, in conjunction with various models for integrating rehabilitation into primary care, including social care where applicable, and cognizant of multimorbidity, could help inform program optimization and scale up initiatives.

### Comprehensiveness

Integrated models of rehabilitation and social care already speak to a more comprehensive offering of rehabilitation beyond those common core components (e.g., physical inactivity, nutrition) strictly related to the underlying risk factors for chronic disease. In scaling the comprehensive nature of rehabilitation, one could argue that human resources are central, including both quantity and quality (e.g., adequate training). Considering social determinants of health and integration within primary care, such human resources may not be restricted to allied health professionals like physiotherapists and also include non-medical human resources (e.g., community champions, community health workers, caregiver-led models). To optimize the human resources required to provide quality rehabilitation for chronic diseases in low-resourced settings, one needs to ask a couple of questions. First, what risk factors are underlying the burden of disease in my specific context, and how may that inform the human resources required (i.e., training needs, composition of the rehabilitation team, integration with social care)? Second, what non-medical human resources are available in my specific context, and how can we optimize program delivery by actively engaging these resources (e.g., task-shifting, self-management)? Importantly, the process of task-shifting in itself may be complex, with important roles for health literacy in self-management models (33) and the community’s social fabric in the feasibility of training lay community workers (34). Thirdly, how can digital innovation be used equitably to promote access, uptake, and/or adherence of technology-driven rehabilitation models, and how does this affect the competencies required of those overseeing such rehabilitation services? The implications thereof, for example, may be that a single physiotherapist oversees a cadre of community health workers for coordinated physiotherapy using a digital telehealth platform rather than solely providing face-to-face clinical services. In this light, we argue that it is pivotal that while we train the next cadre of rehabilitation professionals (35), we embrace the competencies needed to set up and coordinate such task-shifting models as a means to optimize rehabilitation delivery.

### Increasing access

This paper provides an overview of the various critical factors that may be considered in strengthening existing and scale-up of new rehabilitation programs. However, addressing a single factor is unlikely to result in the provision of quality rehabilitation care, as each factor should be recognized as equally important yet equally inadequate if addressed in isolation. We argue that the critical factors, the nine subsystems, in conjunction with existing frameworks (e.g., WHO rehabilitation competency framework) that relate to these subsystems can assist researchers and policymakers in making informed decisions in scaling-up rehabilitation or in conteptualizing the research and information needs. What becomes apparent from this work is that upscaling rehabilitation is complex and requires intersectoral and interdisciplinary impetus across the continuum of care.

## Limitations

The process and sources to identify critical factors that affect the implementation of rehabilitation in low-resource settings can be considered objective and reproducible. Given the call for an intersectoral approach to address the rehabilitation need, involving broader stakeholder groups, which include patients, communities, and government agencies, in identifying the interplay between factors could add to the richness and complexity. Subsequently, such an intersectoral approach may further enhance our understanding of the system within which rehabilitation is to be provided. However, such pre-existing ‘systems’ will be highly context-specific. Hence, we argue that this work should be considered a template of factors that can be considered in scale-up rehabilitation and program optimization, yet that contextualization to any specific situation is required (36).

## Conclusions

The present study highlights the complexity of rehabilitation for people with chronic disease living in low-resource settings. The systems lens provides a framework for researchers and clinicians to foster a rich understanding of the factors influencing the implementation of rehabilitation and the interdependence of the factors within a low-resourced context. The complexity identified encourages a focus on innovative and intersectoral approaches to address the rehabilitation needs in low-resourced settings.
